# Dietary Glutamic Acid Modulates Immune Responses and Gut Health of Weaned Pigs

**DOI:** 10.3390/ani11020504

**Published:** 2021-02-15

**Authors:** Hyunjin Kyoung, Jeong Jae Lee, Jin Ho Cho, Jeehwan Choe, Joowon Kang, Hanbae Lee, Yanhong Liu, Younghoon Kim, Hyeun Bum Kim, Minho Song

**Affiliations:** 1 Division of Animal and Dairy Science, Chungnam National University, Daejeon 34134, Korea; kyounghyunjin@gmail.com (H.K.); leejeongjae@gmail.com (J.J.L.); kangkr99@naver.com (J.K.); 2 Division of Food and Animal Science, Chungbuk National University, Cheongju 28644, Korea; jinhcho@cbnu.ac.kr; 3 Department of Beef Science, Korea National College of Agriculture and Fisheries, Jeonju 54874, Korea; choejhw@gmail.com; 4 Pathway Intermediates, Seoul 06253, Korea; lhb@easybio.co.kr; 5 Department of Animal Science, University of California, Davis, CA 95616, USA; yahliu@ucdavis.edu; 6 Department of Agricultural Biotechnology and Research, Institute of Agriculture and Life Science, Seoul National University, Seoul 08826, Korea; ykeys2584@snu.ac.kr; 7 Department of Animal Resources Science, Dankook University, Cheonan 31116, Korea

**Keywords:** glutamic acid, gut microbiota, ileal gene expression, immune responses, intestinal morphology, nutrient digestibility, weaned pigs

## Abstract

**Simple Summary:**

Weaning stress can lead to intestinal barrier dysfunction, immune system destruction, and intestinal microbiota disruption, thereby reducing the absorption of nutrients and causing intestinal diseases. Glutamic acid is a non-essential amino acid that is abundantly present in the body and plays an essential function in cellular metabolism and immune responses. In this study, the effects of dietary glutamic acid on the growth performance, nutrient digestibility, immune responses, and intestinal health of weaned pigs were evaluated. Based on the results, dietary glutamic acid increased growth performance, nutrient digestibility, intestinal morphology, and ileal gene expression of tight junction proteins of weaned pigs and modified immune responses and gut microbiota. This study provides information to understand the functional use of dietary glutamic acid as a feed additive for improving the growth performance and intestinal health of weaned pigs.

**Abstract:**

Dietary glutamic acid (GLU) is used as a feed additive because of its functional characteristics that may affect the growth performance and health of pigs. This study was carried out to determine the effects of dietary GLU on growth performance, nutrient digestibility, immune responses, and intestinal health of weaned pigs. A total of ninety-six weaned pigs (8.07 ± 1.17 kg of body weight; 28 days of age) were assigned to two dietary treatments (8 pigs/pen; 6 replicates/treatment) in a randomized complete block design (block: body weight): (1) a typical weaner diet (CON) and (2) CON supplemented with 0.5% GLU. The experimental period was for 4 weeks. All data and sample collections were performed at the specific time points during the experimental period. Pigs fed GLU had higher average daily gain and average daily feed intake for the first two weeks and nutrient digestibility than pigs fed CON. In addition, dietary GLU increased villus height to crypt depth ratio, number of goblet cells, and ileal gene expression of claudin family and occludin compared with CON, but decreased serum TNF-α and IL-6 and ileal gene expression of TNF-α. Moreover, pigs fed GLU had increased relative composition of bacterial communities of genus *Prevotella* and *Anaerovibrio* and decreased genus *Clostridium* and *Terrisporobacter* compared with those fed CON. This study suggests that dietary GLU influences growth performance and health of weaned pigs by modulating nutrient digestibility, intestinal morphology, ileal gene expression of tight junction proteins and cytokines, immune responses, and microbial community in the gut.

## 1. Introduction

The gastrointestinal (GI) tract digests and absorbs nutrients and acts as a barrier against toxic compounds and pathogens from the diets taken. Moreover, intestinal epithelial cells are sealed with paracellular tight junctions to regulate the permeability of the intestinal barrier, which is associated with nutrient absorption and immune activity [1]. The epithelial barrier in newborn pigs is rapidly formed with decreased intestinal permeability and optimized liquid-form nutrient absorption during the lactation period. After weaning, piglets undergo several challenges, such as the separation from their mothers, mixing with other piglets, transferring to new environments, switching from digestible liquid diets to solid diets, and immature digestive and immune systems [2,3]. Generally, the weaning stress induces the intestinal dysfunction of weaned pigs, leading to reduced growth performance and increased intestinal problems and diseases that are the major concerns in swine production [4].

It is known that glutamic acid (GLU) is a non-essential amino acid that is abundantly present in the body and plays an essential function in cellular metabolism and immune responses [5]. The GLU is a precursor for protein synthesis and has been attracting attention for its diverse metabolic functions in weaned pigs [6,7]. Moreover, GLU is an important precursor for intestinal formation and immune system in the small intestine and can contribute to the immediate effect on nutrient absorption [7]. Previous studies reported that GLU improved the performance of weaned pigs [7,8,9]. The potential mechanisms of its action are as follows: (1) stimulation of oral and visceral sensory fibers [10], (2) an energy source to form the intestinal barrier structure and functions [11], and (3) regulation of intestinal nervous and immune systems as a signaling compound [12]. However, the connection between dietary GLU and gut physiological benefits under weaning stress is not fully understood. Therefore, the objective of this study was to investigate the effects of dietary GLU on the growth performance, nutrient digestibility, immune responses, and intestinal health of weaned pigs.

## 2. Materials and Methods

The Institutional Animal Care and Use Committee of Chungnam National University reviewed and approved the animal experiment protocol of this study (approval # 201909A-CNU-163). All animal handing and sampling procedures in this study followed the guidelines and regulations for the animal use.

### 2.1. Experimental Design and Diets 

In a randomized complete block design [block: body weight (BW)], a total of ninety-six weaned pigs [(Landrace × Yorkshire) × Duroc; 8.07 ± 1.17 kg of initial BW; 28 days of age] were assigned to two dietary treatments (8 pigs per pen; 6 replicate pens per treatment): (1) a typical weaner diet (CON) and (2) CON supplemented with 0.5% GLU. The experimental period was for 4 weeks. The basal diet was formulated to meet or exceed the nutrient requirements for weaned pigs estimated by the National Research Council (Table 1) [13]. The GLU product was obtained from a commercial supplier (NF Biotechnologies Co., Beijing, China). The pigs had access to feeder and waterer ad libitum and were in the same sized pen (2 m × 2 m) during the entire experimental period. The environmental conditions were automatically controlled by a mechanical system with the ambient temperature maintained at 25–28 °C, and the lighting program regulated on a 12 h light/dark cycle.

### 2.2. Data and Sample Collection 

The pigs’ BW and supplied and residual feeds in each pen were weighed to evaluate growth performance. The average daily gain (ADG), average daily feed intake (ADFI), and feed efficiency (G:F; gain to feed ratio) were calculated from day 1 to 7, day 8 to 14, day 1 to 14, day 15 to 28, and day 1 to 28. The fecal score of pigs in each pen was visually checked with a score range of 1 to 5 (1 = watery diarrhea, 2 = severe diarrhea, 3 = mild diarrhea, 4 = moist feces, and 5 = normal feces) for the first 2 weeks by two independent experimenters [14]. The frequency of diarrhea was calculated by counting pen days with a pen average diarrhea score of less than 3. Blood samples were taken from the jugular vein of the randomly selected 1 pig per pen using 10 mL of tubes with or without ethylenediaminetetraacetic acid (EDTA) on day 1, 7, and 14. The blood samples in the tubes without EDTA were kept at room temperature and centrifuged at 3000× *g* for 15 min at 4 °C to obtain the serum. Serum samples were stored at −20 °C until analysis of immune responses [15]. On the last week of the experiment, chromic oxide (Cr_2_O_3_; Daejung Chemicals & Materials Co. Ltd., Siheung, Gyeonggi, Korea), as an indigestible maker, was mixed with 2 g per kg of diets. After the adaptation period of 4 days, fecal samples were collected from the randomly selected 1 pig per pen by rectal palpation and stored at −20 °C to measure apparent nutrient digestibility [16]. Fecal samples were also obtained from the randomly selected 3 pigs with same BW per treatment on the last day of the experiment by rectal palpation to evaluate their gut microbiota by the metagenome analysis with pyrosequencing method [17]. The collected samples were stored at −80 °C using sterile test tubes prior to metagenome analysis. On the last day, the pigs were anesthetized by 2 mL suxamethonium chloride (Succicholine, Ilsung Pharm. Co. Ltd., Seoul, Korea) via intramuscular injection to the randomly selected 1 pig per pen. After the anesthesia, the pigs were euthanized with CO_2_ gas. Ileal digesta samples were collected between the distal ileum and ileocecal junction and stored at −20 °C until ileal nutrient digestibility analysis. Duodenal, jejunal, and ileal segments were longitudinally cut to about 3 cm and washed with distilled water. The collected intestinal tissues were fastened with 10% formalin solution (neutral buffered) for further intestinal morphology analysis [16]. After washing another ileal tissue with distilled water, the mucosa was scraped using surgical scalpel and stored in liquid nitrogen and then at −80 °C for gene expression analysis.

### 2.3. Nutrient Digestibility Analysis 

For chemical analysis, diets and frozen fecal and ileal digesta samples were dried using the air forced drying oven at 65 °C for 72 h and finely ground by 1 mm screen (Cyclotec 1093; Foss Tecator AB, Hoganas, Sweden). All prepared diets, feces, and ileal digesta samples were analyzed for nutrient digestibility of dry matter (DM; method 930.15), crude protein (CP; method 988.13), and energy by a bomb calorimeter (Model C2000, IKA^®^, Staufen, Germany) based on the Association of Official Analytical Chemists [18]. Chromium concentration was detected by an absorption spectrophotometer (Hitachi Z-5000 Absorption Spectrophotometer, Hitach High-Technologies Co., Tokyo, Japan) to measure nutrient apparent ileal digestibility (AID) and apparent total tract digestibility (ATTD) of weaned pigs. The AID and ATTD of DM, CP, and energy were calculated by the index method based on previous report [19].

### 2.4. Intestinal Morphology Analysis

The procedures for measurements of small intestinal morphology were described by previous report [16]. The prepared intestinal samples were arranged in paraffin and stained with hematoxylin and eosin (H&E). The stained slides of small intestine were scanned using fluorescence microscopy (TE2000, Nikon, Tokyo, Japan) with a charge-coupled device camera (DS-Fil, Nikon, Tokyo, Japan) and processed with NIS-Elements BR software 3.00 (Nikon, Tokyo, Japan). From the stained slides of each section of the intestine, villus height (VH), crypt depth (CD), the ratio between VH and CD (VH:CD), villus width (VW), villus area (VA), and the number of goblet cells were determined by selecting fifteen straight and integrated villi with crypts and goblet cells. 

### 2.5. Ileal Gene Expression Analysis

The ileal gene expressions of tight junction proteins [claudin-1 (CLDN1), claudin-2 (CLDN2), claudin-3 (CLDN3), claudin-4 (CLDN4), and occludin (OCLN)] and mucin-1 (MUC1) and inflammatory cytokines [tumor necrosis factor-α (TNF-α), interleukin-1β (IL-1β), interleukin-6 (IL-6), interferon-gamma (INF-γ), and monocyte chemoattractant protein-1 (MCP1)] were analyzed using quantitative real-time polymerase chain reaction (qRT-PCR). Total RNA was isolated from each sample by HiGene^TM^ total RNA prep kit (BIOFACT, Daejeon, South Korea) and the concentration and quantities of each sample were evaluated by a spectrophotometer (NanoDrop^®^ND-1000; NanoDrop Technologies, Wilmington, DE, USA) after cDNA synthesis by Quantitect^®^ Reverse Transcription kit (Qiagen GmbH, Hilden, Germany). The qRT-PCR analysis was performed by StepOnePlus RT-PCR system (Applied Biosystems, Foster City, CA, USA), SFCgreen^®^ (BIOFACT, Daejeon, Korea), and gene-specific designed primers (Bioneer, Daejeon, Korea). Primer Express^TM^ Software (Applied Biosystems, Foester City, CA, USA) was used to design primer sequences, which are shown as gene-specific forward and reverse sequences in Table 2. The comparative cycle threshold (Ct) value observed using ΔΔCt method (2^−ΔΔCt^) was utilized for the relative quantification of gene expression calculated relative to the values from CON group, and 18S rRNA was used as an internal control for the quantification of target genes [20].

### 2.6. Hematology and Immune Response Analyses

The blood profiles were analyzed from the whole blood samples in EDTA tubes. The number of white blood cells (WBC), red blood cells (RBC), hemoglobin (HGB) and hematocrit (HCT) were measured using an automated hematology analyzer calibrated for porcine blood (scil Vet abc hematology analyzer, scil animal care company, F-67120 Altorf, France). The serum samples were used to analyze the immune responses such as cortisol, TNF-α, transforming growth factor- β1 (TGF-β1), IL-1β, and IL-6 using porcine enzyme-linked immunosorbent assay (ELISA) kits (R&D System Inc., Minneapolis, MN, USA), following the protocols provided by the manufacturer. The results were measured by a microplate reader at 450 nm (Epoch microplate spectrophotometer, BioTek instruments Inc., Winooski, VT). Intra-assay coefficients of variation for cortisol, TNF-α, TGF-β1, IL-1β, and IL-6 were 9.2, 6.9, 2.9, 7.2, and 5.1%, respectively; inter-assay coefficients were 21.2, 9.2, 9.1, 8.7, and 7.4%, respectively.

### 2.7. Gut Microbiota Analysis

Total DNA from the feces of weaned pigs was extracted using the QIAamp DNA Stool Mini Kit (QIAGEN, Hilden, Germany). Gene-specific sequences of targeting region V3-V4 of 16s rRNA microbial genes were amplified with specific primers as listed previously [21]. Sequenced primers were designed based on 16s rRNA metagenomic sequencing library [22]. By polymerase chain reaction (PCR), Bakt 341F (5′-CCTACGGGNGGCWGCAG-3′) and Bakt 805R (5′-GGACTACHVGGGTWTCTAAT-3′) with barcodes were amplified [23]. The PCR product purification was performed by Wizard SV Gel and PCR Clean-Up System (Promega, Fitchburg, WI, USA). The purified PCR product was confirmed by 1% agarose gel electrophoresis. The amplicon sequencing was performed by Illumina MiSeq platform in a commercial company (Macrogen Inc., Seoul, South Korea). In order to analyze raw sequence data, the paired-end reads from next-generation sequencing of barcode and primers were removed from the original sequence. Raw paired-end reads were merged using Fast Length Adjustment of Short reads (FLASH, v 1.2.11), a rapid and accurate software tool [24] with a 7 cycle index read (Macrogen Inc., Seoul, South Korea). A total of 150,150 ± 10,338 average assembly sequences reads were obtained. The following step was denoising and clustering using CD-HIT-OTU to find an operational taxonomic units (OTUs) defined at an identity cut-off of 97% [25]. Bacterial taxonomic composition and community for each sample were performed using UCLUST [26] and Quantitative Insights into Microbial Ecology (QIIME) [27] based on ribosomal database project classifier for 16S rRNA. Alpha diversity was calculated using OTUs, Chao 1, Shannon, and Inverse Simpson, with estimated over 0.99 Good’s coverage by QIIME. The principal coordinate analysis (PCoA) of beta diversity was performed by UniFrac distance metrics in QIIME to relate the bacterial community between dietary treatments. The taxonomic composition of each sample at phylum and genus levels was shown as a percentage based on relative abundance.

### 2.8. Statistical Analyses

The data were analyzed by the General Linear Model procedure of SAS (SAS Inst. Inc., Cary, NC, USA) using the PDIFF option in a randomized completely block design (block: BW). The experimental unit was the pen. The statistical model for growth performance, blood profiles, nutrient digestibility, intestinal morphology, and immune responses included effects of dietary treatment as main effects and BW as a covariate. The Chi-square test was used for the frequency of diarrhea. The t-test was used for the comparison of ileal gene expression of tight junction proteins and cytokines between dietary treatments. The STAMP and Prism software (Prism 5.00, GraphPad Software, La Jolla, CA, USA) as well as MicrobiomeAnalyst (https://www.microbiomeanalyst.ca/, (accessed on 22 January 2021)) were used for the alpha diversity and taxonomic classification as well as the beta diversity of microbial populations, respectively, between dietary treatments. Statistical significance and tendency between dietary treatments were considered at *p* < 0.05 and 0.05 ≤ *p* < 0.10, respectively. 

## 3. Results

### 3.1. Growth Performance and Nutrient Digestibility

Dietary GLU increased ADG and ADFI of weaned pigs from day 8 to 14 (*p* < 0.05) as well as from day 1 to 14 (*p* < 0.10) compared with CON (Table 3). However, there were no differences on growth performance of weaned pigs from day 15 to 28 and during overall experimental period and frequency of diarrhea for the first two weeks after weaning between dietary treatments (Table 3). Pigs fed GLU had significantly increased (*p* < 0.05) AID and ATTD of DM and energy compared with those fed CON (Table 4).

### 3.2. Intestinal Morphology and Ileal Gene Expression of Tight Junction Proteins and Cytokines

Pigs fed GLU had higher VH (*p* < 0.10), VH:CD (*p* < 0.05), and number of goblet cells (*p* < 0.05) in duodenum, VH:CD (*p* < 0.10) and number of goblet cells (*p* < 0.05) in jejunum, and VH:CD (*p* < 0.10), VA (*p* < 0.05), and number of goblet cells (*p* < 0.10) in ileum than those fed CON (Table 5). However, there were no differences in CD and VW between dietary treatments (Table 5).

**Figure 1:**
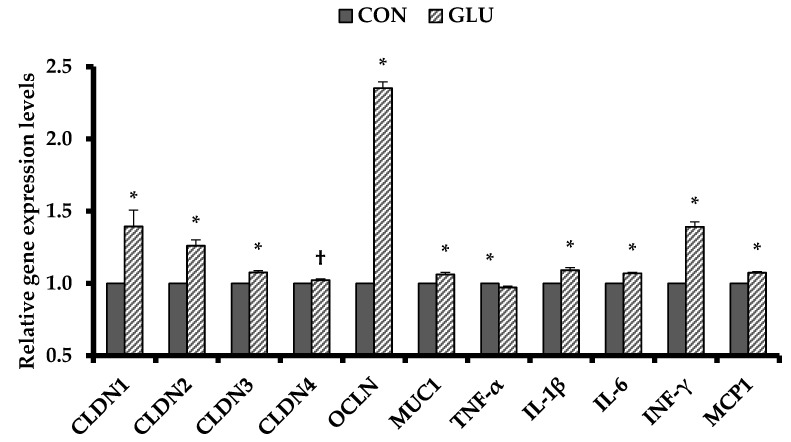
Relative ileal gene expression levels of tight junction proteins and inflammatory cytokines of weaned pigs. Each value is the main value of 6 replicates (1 pig/pen). CON = a typical weaner diet; GLU = CON with 0.5% glutamic acid; CLDN1 = claudin-1; CLDN2 = claudin-2; CLDN3 = claudin-3; CLDN4 = claudin-4; OCLN = occludin; MUC1 = mucin 1; TNF-α = tumor necrosis factor-α; IL-1β = interleukin-1β; IL-6 = interleukin-6; INF-γ = interferon-γ; MCP1 = monocyte chemoattractant protein-1. Data were analyzed by t-test. *^,†^ Different between CON and GLU (*, *p* < 0.05; ^†^, *p* < 0.10).

Dietary GLU increased (*p* < 0.05) ileal gene expressions of claudin family (CLDN1, 2, and 3) and OCLN as tight junction proteins and MUC1 and inflammatory cytokines (IL-1β, IL-6, INF-γ, and MCP1) compared with CON (Figure 1). However, pigs fed GLU had decreased (*p* < 0.05) ileal gene expression of TNF-α compared with those fed CON (Figure 1).

### 3.3. Blood Profiles and Immune Responses

As shown in Table 6, dietary GLU did not influence WBC, RBC, and HCT compared with CON. Interestingly, pigs fed GLU tended to have higher (*p* < 0.10) HGB on day 7 than those fed CON. As shown in Table 7, pigs fed GLU tended to have lower (*p* < 0.10) serum TNF-α on day 7 and 14, serum IL-6 on day 7, and IL-1β on day 14 compared with those fed CON. 

### 3.4. Gut Microbiota

**Figure 2:**
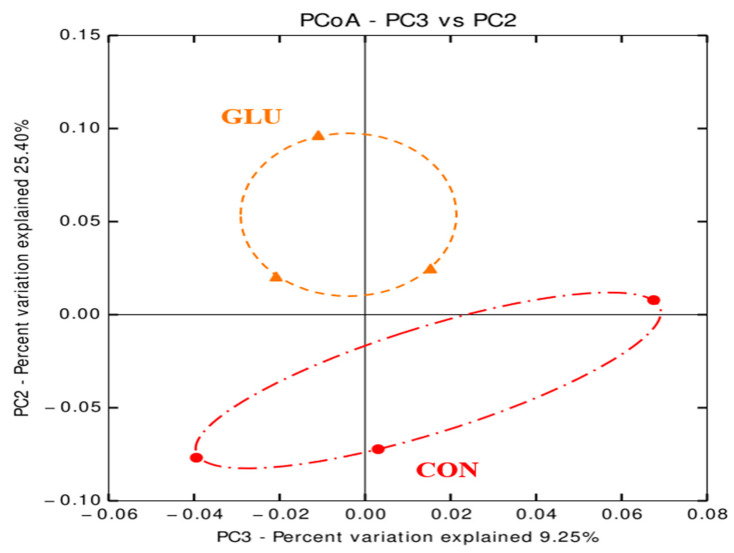
Discriminant analysis of the principal components of fecal microbiota (*n* = 3). The six differently abundant bacteria genera represent the number of variables in the model. Individual pig samples for treatments are designated with the following symbols: CON = a typical weaner diet (red, ●), GLU = CON with 0.5% glutamic acid (orange, ▲). R-value = 0.678, *p*-value < 0.05.

**Figure 3:**
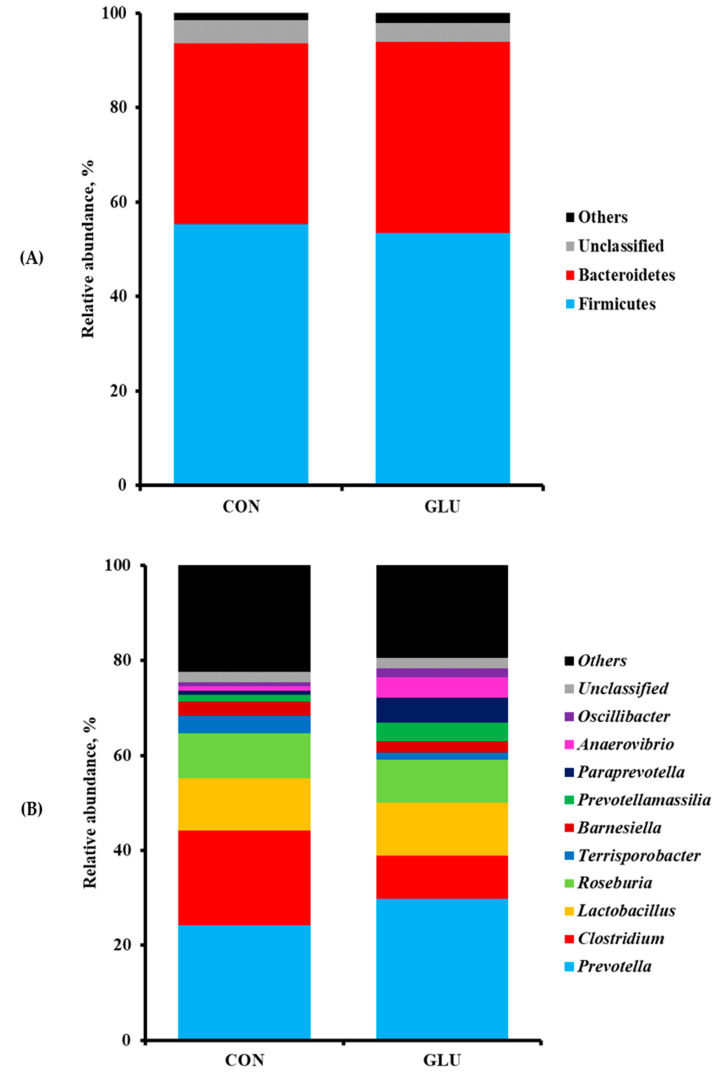
Taxonomic classification of total bacteria at phylum (**A**) and genus (**B**) levels retrieved from pooled DNA amplicons for each treatment group (*n* = 3). The proportion of fecal microbiota of weaned pigs less than 1% was not included. CON = a typical weaner diet; GLU = CON with 0.5% glutamic acid.

The numbers of sequence reads obtained from weaned pigs were 145,258 and 155,043 for CON and GLU, respectively, after quality filtering, but the alpha diversity indexes, such as OTUs, Chao1, Shannon, and Inverse Simpson, were not different between dietary treatments (Table 8). The PCoA plot for the beta diversity was shown in Figure 2. The distinct separation of microbial populations was visually found between CON and GLU. The taxonomic classification of total bacteria at the phylum and genus levels between dietary treatments are shown in Figure 3. The microbiota of weaned pigs consisted of more than 90% of Firmicutes and Bacteroidetes at the phylum level in both dietary treatments, but there was no difference in microbiota composition at the phylum level between dietary treatments (Figure 3A). At the genus level, the relative abundances of *Prevotella* and *Anaerovibrio* were increased (*p* < 0.05) in weaned pigs fed GLU compared to those fed CON (Figure 3B). However, the relative abundances of *Clostridium* and *Terrisporobacter* were lower (*p* < 0.05) in weaned pigs fed GLU than in those fed CON (Figure 3B). 

## 4. Discussion

The post-weaning period is critical for the intestinal development of weaned pigs, as weaning stress can lead to intestinal barrier dysfunction, immune system destruction, and intestinal microbiota disruption, thereby reducing the absorption of nutrients and causing intestinal diseases such as diarrhea [4]. Therefore, appropriate nutrient supply for weaned pigs is one of essential factors to improve the intestinal health. Several studies reported that some feed additives, such as plant extracts, exogenous enzymes, and GLU, had beneficial effects on growth performance and gut barrier functions of pigs [8,14,15,28]. In particular, GLU is regarded as one of the most promising performance-enhancing additives with amino acid synthesis properties [8]. However, the correlations of dietary GLU on growth performance and intestinal barrier and immune functions of pigs are not fully understood. In the present study, we demonstrated that dietary GLU efficiently influenced growth rate and gut health of weaned pigs.

The present study showed dietary GLU enhanced the ADG and ADFI of weaned pigs for the first two weeks after weaning. These observations are in agreement with previous studies, suggesting that dietary GLU may give more beneficial effects on growth performance of weaned pigs around the first 10 to 21 days after weaning [9,29,30]. These results indicate that dietary GLU promotes the growth performance of pigs by increasing feed intake and performing as a metabolic fuel that more effectively increases cell turnover rate during intestinal development [8,9]. Moreover, it is well known that GLU has umami properties that may have an important role in the taste, palatability, and acceptability of the feeds [6,31]. The GLU not only stimulates taste and oral sensation by binding with umami receptors in the oral cavity, but is also involved in signaling in the brain for the autonomic reflex of the digestive responses associated with salivation for chewing and swallowing [11]. In the present study, there was no dietary GLU effect on the frequency of diarrhea during the first two weeks after weaning that may be caused by the weaning events. However, a previous study reported that supplementation with 4% dietary GLU in weaner diets alleviated diarrhea in the *Escherichia coli* infected pigs [32]. This is because the metabolites that produced by GLU, such as N-acetyl glucosamine and N-acetylgalactosamine residues, bind to same receptors for *Escherichia coli* in intestinal epithelial cells to relieve diarrhea [33]. These findings suggested that more nutrient supply is required for the growth of weaned pigs in early intestinal development and dietary GLU may be involved in amino acid metabolisms in the initial phase [32,33].

A previous study reported that several stress factors, such as maternal separation, mixing/crowding, dietary change, and GI infection after weaning, could cause intestinal dysfunction which inhibits the absorption of nutrients in the small intestine [34]. During the weaning period, the intestinal structure and function of pigs are immature by declining the reduction in villus enzyme activity, VH, and transepithelial electrical resistance [35]. In the results from the present study, dietary GLU enhanced the AID and ATTD of DM and energy. These results are in agreement with previous studies, suggesting that GLU improves the intestinal digestive process by acting as a signaling compound in the intestinal nervous system and regulating the neuroendocrine functions of the GI tract [11,36]. The GLU-related receptors, such as T1R1, T1R3, mGluR4 and mGluR1, are distributed in the stomach and intestine, regulating the mucosal responses in the stomach and duodenum [36]. Moreover, GLU is involved in maintaining the intestinal barrier functions and structure of weaned pigs. The present study also observed that dietary GLU improved the intestinal morphology by increasing VH:CD and number of goblet cells. These observations are in agreement with several in vivo and in vitro studies, reporting that GLU regulates the proliferation and differentiation of intestinal epithelial cells [36,37,38]. The GLU serves as an important energy source for cell division in porcine mucosa and inhibits the activities of apoptotic proteins, such as caspase families, phospho-p53, and cytochrome, which induce intestinal cell apoptosis in weaned pigs [7]. Furthermore, the intestinal barrier functions in the intestinal epithelium play a critical role in nutrient absorption by pump action via the enzyme activity of the electrolyte transporters distributed on the brush border. In addition, the tight junction between the epithelial cells regulates gate function through intercellular membrane proteins, such as ZO-1, occludin, and claudins [34]. Similarly, the present study verified that dietary GLU improved the intestinal barrier functions by improving gene expressions of tight junction proteins in the ileal tissues of weaned pigs. These results are in agreement with previous studies on nutrient digestibility and gut barrier functions [8,39,40]. These physiological benefits suggest that dietary GLU improved nutrient digestibility directly by strengthening the intestinal barrier functions.

Weaning stress overreacts the hypothalamic pituitary adrenal axis and corticotropin releasing factor system to release mast cell proteases and TNF-α in the intestine, which increase intestinal epithelium permeability and induce the penetration of external antigens [34]. Inflammatory cytokines play the most important part in the regulation of immune system and are closely related to weaning stress [4]. In the present study, dietary GLU decreased TNF-α, IL-1β, and IL-6 levels in the serum and downregulated TNF-α gene expression levels in the ileal tissue, suggesting that dietary GLU may have anti-inflammatory effects. Previous studies indicated that GLU stimulated the synthesis of intestinal mucosa cells and might be used as major fuel and substrates for metabolism and immune responses [5,7,41]. Like other immune response-related amino acids, GLU also plays a role in regulating the immune system. Previous studies showed that dietary GLU alleviated the incidence of diarrhea and had anti-oxidative stress effects in weaned pigs [42,43,44]. The results from the present study are consistent with these previous observations. Therefore, the immune responses by dietary GLU in weaner diets supported that dietary GLU might contribute to improvement of the intestinal health and development during the weaning stress.

Weaning is a major challenge for piglets to adapt to the GI microbial colonization and physiological changes in their intestine, following the transition of diets from milk to feed as well as other changes. Therefore, the gut microbial analysis was performed as a pilot study using small numbers of animals to assess whether dietary GLU changed microbial population of weaned pigs. Our results showed that the phyla Firmicutes and Bacteroidetes comprised over 90% of all bacteria in the microbiomes of pigs in both CON and GLU. Previous studies reported that both phyla Firmicutes and Bacteroidetes contributed to the production of volatile fatty acids, such as acetate, propionate, and butyrate, through the digestible fiber in plant-derived weaning diets compared with the pre-weaning milk diets [45,46]. In addition, higher proportions of bacteria from both phyla Firmicutes and Bacteroidetes were associated with an increase in BW and these phyla improved the energy production capacity of pigs during the digestion process [47].

The present study also showed pigs fed GLU had more *Prevotella* and *Anaerovibrio* than those fed CON. The previous study mentioned that *Prevotella* was more common in the gut microbiota of pigs after weaning than in that of breast-fed piglets because they could ferment the indigestible polysaccharides into short-chain fatty acids in the gut [17]. In addition, *Anaerovibrio* strains, such as *A. lipolytica*, are linked to fat metabolism with the production of lipase to hydrolyze triglycerides [48]. In the present study, the relative abundances of genera *Clostridium* and *Terrisporobacter* were also lower in the gut microbiota of weaned pigs fed GLU than in that of weaned pigs fed CON. Generally, spore-forming *Clostridium* strains are recognized as the primary cause of diarrhea in neonatal and weaned piglets [49]. Previous studies reported that the spores and toxins from *C. difficile* and *C. perfringens* originated during the lactation period induced high rates of diarrhea and mortality after weaning [49,50,51]. Moreover, *Terrisporobacter* produces the urinary toxin, such as trimethylamine-*N*-oxide, which is associated with oxidative stress and inflammation in the gut of weaned pigs [52]. Therefore, it is plausible that the increased abundances of *Prevotella* and *Anaerovibrio* in the gut microbiota of weaned pigs fed GLU may contribute to the reduced abundances of *Clostridium* and *Terrisporobacter* compared with those of weaned pigs fed CON, thereby improving the intestinal health of weaned pigs by stabilizing the immune state and intestinal environment.

## 5. Conclusions

The findings of this study demonstrated that dietary GLU had positive effects on the growth performance, nutrient digestibility, immune responses, and intestinal health of weaned pigs. Our data indicate that the integrated enhancements of growth performance and nutrient digestibility of weaned pigs may be associated with improved gut morphology and intestinal barrier functions through the upregulation of tight junction proteins and changed gut microbiota. In addition, our results suggest that dietary GLU modulates serum immune responses and ileal gene expressions of cytokines that may be related to intestinal immune system. Based on these results, supplementation of dietary GLU in weaner diets can provide pigs with beneficial effects on the growth performance and gut health of weaned pigs.

## Figures and Tables

**Table 1:** Composition of the basal diet of weaned pigs (as-fed basis) ^1^.

Item	Basal Diet
Ingredient, %	
Corn (8%)	53.90
Soybean meal (44%)	15.00
Soy protein concentrate	7.50
Whey powder (12%)	12.50
Soybean oil	2.30
Spray dried porcine plasma	2.50
Fish meal, combined	3.00
Limestone	1.20
Monocalcium phosphate	0.80
Vitamin-Mineral premix ^2^	0.40
L-lysine-HCl	0.35
DL-Methionine	0.15
L-Threonine	0.10
Zinc oxide	0.30
Total	100.00
Calculated energy and nutrient contents	
Metabolizable energy, kcal/kg	3400.00
Crude protein, %	21.69
Calcium, %	0.89
Phosphorus, %	0.68
Lysine, %	1.55

^1^ The 0.5% glutamic acid (GLU) was added in the basal diet and replaced by 0.5% corn to make the GLU treatment. GLU, NF Biotechnologies Co., Beijing, China; Chromic oxide, Daejung Chemicals & Materials Co. Ltd., Siheung, Gyeonggi, South Korea. ^2^ Vitamin–mineral premix provided the following quantities of vitamin–mineral per kilogram of basal diet: vitamin A, 12,000 IU; vitamin D_3_, 2500 IU; vitamin E, 30 IU; vitamin K_3_, 3 mg; _D_-pantothenic acid, 15 mg; nicotinic acid, 40 mg; choline, 400 mg; vitamin B_12_, 12 μg; Fe, 90 mg from iron sulfate; Cu, 8.8 mg from copper sulfate; Zn, 100 mg from zinc oxide; Mn, 54 mg from manganese oxide; I, 0.35 mg from potassium iodide; Se, 0.30 mg from sodium selenite.

**Table 2:** Gene specific primer sequences for the gene expression of tight junction proteins and inflammatory cytokines in ileal tissue.

Item ^1^	Forward (5′–3′)	Reverse (5′–3′)
CLDN1	AGAAGATGCGGATGGCTGTC	CCCAGAAGGCAGAGAGAAGC
CLDN2	TCCTCCCTGTTCTCCCTGATAG	CCTTGCAGTGGGCAGGAA
CLDN3	GATGCAGTGCAAAGTGTACGA	GTCCTGCACGCAGTTGGT
CLDN4	TATCATCCTGGCCGTGCTA	CATCATCCACGCAGTTGGT
OCLN	GGAGTGATTCGGATTCTGTCTATGCT	CGCCTGGGCTGTTGGGTTGA
MUC1	CCCTGGCCATCATCTATGTC	TGCCCACAGTTCTTTCGTC
TNF-α	CTTGGGTTTGGATTCCTGGAT	CTTCCCTGGCAGCCACAT
IL-1β	GCCCTGTACCCCAACTGGTA	CCCAGGAAGACGGGCTTT
IL-6	GCGCAGCCTTGAGGATTTC	CCCAGCTACATTATCCGAATGG
INF-γ	GAGCCAAATTGTCTCCTTCTAC	CGAAGTCATTCAGTTTCCCAG
MCP1	TCCCACACCGAAGCTTGAAT	CACAGGAGGGCTGCAGAGA
18S rRNA	GGCTACCACATCCAAGGAAG	TCCAATGGATCCTCGCGGAA

^1^ CLDN1 = claudin-1; CLDN2 = claudin-2; CLDN3 = claudin-3; CLDN4 = claudin-4; OCLN = occludin; MUC1 = mucin 1; TNF-α = tumor necrosis factor-α; IL-1β = interleukin-1β; IL-6 = interleukin-6; INF-γ = interferon-γ; MCP1 = monocyte chemoattractant protein-1.

**Table 3:** Effects of dietary treatments on growth performance of weaned pigs ^1^.

Item ^2^	CON	GLU	SEM	*p*-Value
Day 1 to 7				
Initial BW, kg	8.07	8.06	0.50	0.996
Final BW, kg	9.26	9.22	0.51	0.952
ADG, g/d	170.00	165.71	17.36	0.779
ADFI, g/d	255.07	263.42	18.77	0.760
G:F, g/g	0.666	0.629	0.03	0.305
Day 8 to 14				
Initial BW, kg	9.26	9.22	0.51	0.952
Final BW, kg	12.68	13.22	0.60	0.538
ADG, g/d	488.57	571.43	14.48	0.010
ADFI, g/d	609.76	670.66	16.17	0.024
G:F, g/g	0.801	0.852	0.02	0.156
Day 1 to 14				
Initial BW, kg	8.07	8.06	0.50	0.996
Final BW, kg	12.68	13.22	0.60	0.538
ADG, g/d	329.29	368.57	12.43	0.066
ADFI, g/d	432.42	467.04	15.18	0.093
G:F, g/g	0.761	0.789	0.02	0.412
Day 15 to 28				
Initial BW, kg	12.68	13.22	0.60	0.538
Final BW, kg	19.82	20.00	0.75	0.866
ADG, g/d	510.00	484.29	16.19	0.285
ADFI, g/d	862.34	884.75	34.71	0.658
G:F, g/g	0.591	0.547	0.02	0.125
Day 1 to 28				
Initial BW, kg	8.07	8.06	0.50	0.996
Final BW, kg	19.82	20.00	0.75	0.866
ADG, g/d	419.64	426.43	12.88	0.728
ADFI, g/d	647.38	675.90	24.62	0.372
G:F, g/g	0.648	0.631	0.01	0.189
Frequency of diarrhea ^3^, %	11.90	10.71		0.828

^1^ Each value is the mean value of 6 replicates (8 pigs/pen). ^2^ CON = a typical weaner diet; GLU = CON with 0.5% glutamic acid; SEM = standard error of the mean; BW = body weight; ADG = average daily gain; ADFI = average daily feed intake; G:F = gain to feed ratio. ^3^ Frequency of diarrhea for the first two weeks after weaning (%) = (number of diarrhea with score less than 3/number of pen days) × 100. Data were analyzed using the Chi-square test.

**Table 4:** Effects of dietary treatments on nutrient digestibility of weaned pigs ^1^.

Item ^2^	CON	GLU	SEM	*p*-Value
Apparent ileal digestibility, %				
Dry matter	79.79	82.66	0.32	0.001
Crude protein	73.04	77.77	3.10	0.305
Energy	75.54	80.27	0.92	0.005
Apparent total tract digestibility, %				
Dry matter	84.09	86.91	0.56	0.005
Crude protein	76.83	75.40	0.99	0.333
Energy	81.40	85.61	0.80	0.004

^1^ Each value is the mean value of 6 replicates (1 pig/pen). ^2^ CON = a typical weaner diet; GLU = CON with 0.5% glutamic acid; SEM = standard error of the mean.

**Table 5:** Effects of dietary treatments on intestinal morphology of weaned pigs ^1^.

Item ^2^	CON	GLU	SEM	*p*-Value
Duodenum				
Villus height, μm	310.31	360.24	16.31	0.056
Crypt depth, μm	258.57	245.59	8.86	0.325
VH:CD, μm/μm	1.20	1.47	0.05	0.004
Villus width, μm	137.26	156.97	10.43	0.211
Villus area, μm^2^	38783	46556	3248	0.122
Goblet cells, n	9.65	14.11	0.96	0.008
Jejunum				
Villus height, μm	296.21	336.73	20.55	0.193
Crypt depth, μm	264.07	250.34	24.40	0.699
VH:CD, μm/μm	1.12	1.35	0.08	0.096
Villus width, μm	156.94	163.87	9.35	0.612
Villus area, μm^2^	40935	41516	1781	0.822
Goblet cells, n	9.19	14.40	1.00	0.004
Ileum				
Villus height, μm	390.37	410.01	11.72	0.264
Crypt depth, μm	259.09	240.21	14.05	0.365
VH:CD, μm/μm	1.51	1.71	0.07	0.082
Villus width, μm	142.62	155.88	7.69	0.251
Villus area, μm^2^	30955	38424	1534	0.006
Goblet cells, n	14.98	17.97	1.01	0.072

^1^ Each value is the mean value of 6 replicates (1 pig/pen). ^2^ CON = a typical weaner diet; GLU = CON with 0.5% glutamic acid; SEM = standard error of the mean; VH:CD = villus height to crypt depth ratio.

**Table 6:** Effects of dietary treatments on blood profiles of weaned pigs ^1^.

Item ^2^	CON	GLU	SEM	*p*-Value
White blood cell, ×10^3^/μL				
Day 1	12.87	13.75	2.32	0.793
Day 7	14.83	16.12	1.01	0.392
Day 14	21.83	22.52	1.80	0.794
Red blood cell, ×10^6^/μL				
Day 1	6.00	6.46	0.28	0.279
Day 7	5.88	6.31	0.25	0.252
Day 14	6.46	6.51	0.21	0.865
Hemoglobin, g/dL				
Day 1	10.12	11.32	0.60	0.188
Day 7	8.32	11.38	1.16	0.092
Day 14	11.32	11.43	0.49	0.869
Hematocrit, %				
Day 1	29.65	33.85	1.89	0.148
Day 7	29.03	32.73	1.75	0.165
Day 14	33.38	33.73	1.41	0.865

^1^ Each value is the mean value of 6 replicates (1 pig/pen). ^2^ CON = a typical weaner diet; GLU = CON with 0.5% glutamic acid; SEM = standard error of the mean.

**Table 7:** Effects of dietary treatments on immune responses of weaned pigs ^1^.

Item ^2^	CON	GLU	SEM	*p*-Value
Cortisol, ng/mL				
Day 1	45.42	44.89	3.66	0.921
Day 7	47.91	46.74	1.04	0.447
Day 14	50.47	48.08	2.11	0.442
Tumor necrosis factor-α, pg/mL				
Day 1	64.56	56.77	5.08	0.314
Day 7	132.42	86.86	15.17	0.060
Day 14	127.48	100.53	9.87	0.082
Transforming growth factor-β1, pg/mL				
Day 1	1353.94	1113.66	117.13	0.191
Day 7	1179.26	1199.74	93.12	0.880
Day 14	1106.56	1117.49	43.31	0.863
Interleukin-1β, pg/mL				
Day 1	24.36	25.94	3.77	0.775
Day 7	37.83	37.62	5.31	0.979
Day 14	36.09	24.05	3.61	0.050
Interleukin-6, pg/mL				
Day 1	138.16	135.83	2.25	0.485
Day 7	148.66	138.28	3.73	0.077
Day 14	143.70	134.53	3.58	0.101

^1^ Each value is the mean value of 6 replicates (1 pig/pen). ^2^ CON = a typical weaner diet; GLU = CON with 0.5% glutamic acid; SEM = standard error of the mean.

**Table 8:** Effects of dietary treatments on the alpha diversity of bacteria of weaned pigs ^1^.

Item ^2^	CON	GLU	*p*-Value
Number of sequence reads	145,258 ± 29,513	155,043 ± 25,697	0.687
OTUs	293.00 ± 60.53	281.30 ± 8.14	0.771
Chao1	323.00 ± 63.46	330.30 ± 33.79	0.870
Shannon	5.41 ± 0.67	5.26 ± 0.42	0.758
Inverse Simpson	0.94 ± 0.03	0.93 ± 0.04	0.857

^1^ Each value is the mean value of 3 replicates (1 pig/pen) and presented as mean ± standard deviation. ^2^ CON = a typical weaner diet; GLU = CON with 0.5% glutamic acid; OTUs = operation taxonomic units; Chao1 = richness estimates for an OUT definition; Shannon = account the number and evenness of species; Inverse Simpson = probability that two randomly selected individuals in the habitat belong to the same species.

## Data Availability

The data presented in this study are available from the corresponding author on request.
